# Dry Immersion as a Ground-Based Model of Microgravity Physiological Effects

**DOI:** 10.3389/fphys.2019.00284

**Published:** 2019-03-27

**Authors:** Elena Tomilovskaya, Tatiana Shigueva, Dimitry Sayenko, Ilya Rukavishnikov, Inessa Kozlovskaya

**Affiliations:** ^1^RF SSC – Institute of Biomedical Problems, Russian Academy of Sciences, Moscow, Russia; ^2^Center for Neuroregeneration, Houston Methodist Research Institute, Houston, TX, United States

**Keywords:** dry immersion, motor control, gravity unloading, support withdrawal, supportlessness

## Abstract

Dry immersion (DI) is one of the most widely used ground models of microgravity. DI accurately and rapidly reproduces most of physiological effects of short-term space flights. The model simulates such factors of space flight as lack of support, mechanical and axial unloading as well as physical inactivity. The current manuscript gathers the results of physiological studies performed from the time of the model’s development. This review describes the changes induced by DI of different duration (from few hours to 56 days) in the neuromuscular, sensory-motor, cardiorespiratory, digestive and excretory, and immune systems, as well as in the metabolism and hemodynamics. DI reproduces practically the full spectrum of changes in the body systems during the exposure to microgravity. The numerous publications from Russian researchers, which until present were mostly inaccessible for scientists from other countries are summarized in this work. These data demonstrated and validated DI as a ground-based model for simulation of physiological effects of weightlessness. The magnitude and rate of physiological changes during DI makes this method advantageous as compared with other ground-based microgravity models. The actual and potential uses of the model are discussed in the context of fundamental studies and applications for Earth medicine.

## Introduction

Observations performed after space flights of different durations have shown that weightlessness causes body deconditioning, which is demonstrated by changes in most of the physiological systems (Gazenko et al., 1986; Kornilova, 1987; Kozlovskaya et al., 1988, 1990; Berger et al., 1992; Grigoriev and Egorov, 1992; Edgerton and Roy, 1996; Reschke et al., 1998; Clement et al., 2003). The necessity to study the mechanisms of hypogravitational effects on the human body led to the exploration of experimental models of weightlessness.

Immersion into a liquid analogous by density to human body tissues (Gazenko et al., 1972; Shulzhenko and Vill-Villiams, 1975) and antiorthostatic hypokinesia (head down tilt bedrest, HDBR) (Kakurin, 1968; Genin and Sorokin, 1969) have been proposed to be the most appropriate environment for the physiological studies of hypogravity effects.

Support withdrawal, local load elimination and the proximity of biomechanical conditions of motor activity organization to those in weightlessness from the beginning of space era determined that immersion would be chosen as the best model for training of working operations in weightlessness.

In the early 1960s, scientists started to study the physiological effects of immersion to determine its capacity to simulate the effects of weightlessness (Beckman et al., 1961; Graveline et al., 1961). It has been shown that immersion reproduces microgravity-induced changes in the human body’s motor (Ovsyannikov, 1972) and cardio-vascular (Shulzhenko and Vill-Villiams, 1976) systems and affects other physiological functions. The usage of immersion as a valuable model was limited only by discomfort and possible risks of long term skin contact with the liquid environment.

## History of DI Model Development

**FIGURE 1:**
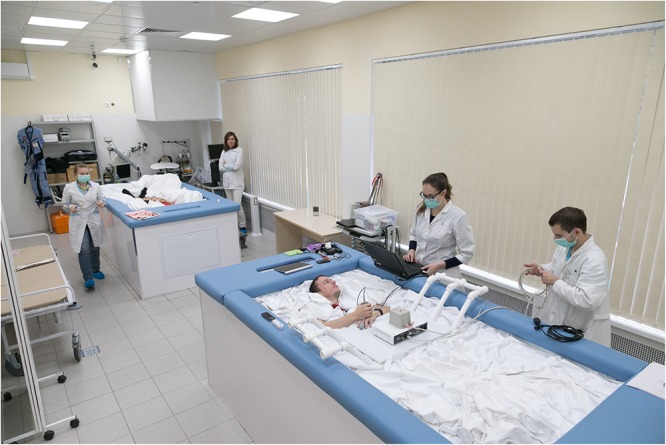
Overall view of dry immersion facilities at IBMP. Image credit IBMP/Oleg Voloshin.

At the beginning of the 1970s two investigators from the Institute of Biomedical Problems (Russia), K. B. Shulzhenko and I. F. Vil-Villiams, developed a method of long term immersion, dry immersion (DI), with the use of special waterproof and highly elastic fabric (Shulzhenko, 1975; Shulzhenko and Vill-Villiams, 1975). A test subject wearing a shirt and trunks was put on waterproof fabric and immersed into a deep bath up to the neck level, in a supine position. The area of the fabric’s surface considerably exceeded the area of the water surface. The folds of the waterproof fabric allowed the person’s body to be enveloped from all sides freely (Figure 1).

The high elasticity properties of the fabric artificially created conditions similar to zero gravity via floatation. Thus, DI reproduced three effects of weightlessness: physical inactivity, support withdrawal and elimination of the vertical vascular gradient. Since its development, DI has been the main model in Russia for studying the effects of weightlessness lasting 5–7 days, similar to the duration of short-term flights on orbital space stations.

Most of the results described in this review come from studies where healthy male volunteers were placed, individually or in pairs, in a supine position in a bath with dimensions of 200 × 100 × 100 (300 × 300 × 200) cm. The bath was filled with water at a temperature that was kept constant at 33 ± 0.5°C. The daily routine was specified in accordance with the schedule of studies, countermeasure procedures (if the experiment included them), including 8 h of sleep, 3–4 meals, a medical supervision program and experimental studies. The research participants were taken out of DI for 15–20 min each day for sanitary and hygienic procedures with the usage of a special lift rising from the bottom of the bath.

In 1974, E. B. Shulzhenko and I. F. Vil-Villiams performed the longest 56-day DI experiment with two volunteers, which convincingly proved the applicability and safety of the DI model for reproducing long-term microgravity effects. The results of this experiment showed that 56 days of DI decreased the redundant capabilities of the blood circulation system, providing deconditioning of the body as a whole. The changes of the functional state of the blood circulation system demonstrated signs of an adaptation processes with the predominance of parasympathetic tone and decline of resistance to g-loading in the “head-hip” direction and low body negative pressure (Shulzhenko and Vill-Villiams, 1976; Vil-Villiams and Shulzhenko, 1978).

In early years of the DI’s use, the main direction of research was a description of its effects on different functions of the human body. Attention of the researchers was primarily focused on vital functions, including the activities of the cardiovascular and respiratory systems and metabolism. These studies revealed similarities in the depth, dynamics and direction of DI-induced physiological changes to those observed in space flights (Kamenskiy et al., 1976; Aleksandrova et al., 1980; Fomin, 1981; Ivanov et al., 1983; Kozinets et al., 1983; Orlov, 1985; Yarullin et al., 1987; Genin and Galichiy, 1995; Mikhailov et al., 1995).

It has been shown that the subjective experience of exposure to DI at its initial stage is perceived as a comfortable and pleasant state of relaxation (Shulzhenko and Vill-Villiams, 1976; Shulzhenko et al., 1983a; At’kov and Bednenko, 1987; Navasiolava et al., 2011a; Tomilovskaya, 2013a,b). However, heaviness in the head and nasal congestion in some cases were observed later (presumably due to a redistribution of the body’s fluids in the cranial direction). During the first 2–3 days of DI, most of test subjects reported the development of abdominal pain (Rukavishnikov et al., 2014b) and back pain of differing intensity (Rukavishnikov et al., 2017a,b); on the 3rd or 4th day of DI, all of these signs disappeared. Sleep disorders (mostly due to back and abdominal pain), loss of appetite, meteorism and constipation are also observed in the initial stages of DI (Shulzhenko, 1975; Fomin, 1981; Shulzhenko et al., 1984; Tomilovskaya, 2013a,b). Under conditions of “double baths,” when 2 subjects shared the same water pool, body movements of one subject were sometimes followed by the symptoms of space motion sickness for the other one (Barmin et al., 1983; Struhal et al., 2002; Kreidich et al., 2007), similar to those registered during the 1st days of space flight (Kornilova, 1997; Williams et al., 2009) and bedrest (Greenleaf, 1984; Baum and Essfeld, 1999).

In the 1970s the water immersion was used broadly as a fast and convenient model for assessing the efficacy of countermeasure means and methods. Those studies were performed at the Russian Scientific Testing Institute of Aviation and Space Medicine by A. M. Genin, I. D. Pestov, V. I. Stepantsov, A. V. Eremin and other specialists of space mission medical support. Later, this research was entrusted to the newly created Institute of Biomedical Problems. However, the impossibility of long term stay in the water immersion imposed serious restrictions to the range of tested countermeasure means. The DI method opened the possibility for long-duration exposures and started a new line of studies.

## Similarity of DI- and Hdbr-Induced Effects

In parallel to the DI experiments, HDBR research has been developing and the model has achieved worldwide popularity (Watenpaugh, 2016). This is not surprising because the organization of HDBR is relatively easier than DI, since the test subjects are available for the studies for almost 24 h a day at all the stages of experiment. Comparison of the data has shown similarity of the changes observed in these two models (Aleksandrova et al., 1980; Chaika et al., 1982; Grigoriev et al., 2004). However, the dynamics and the depth of these changes significantly differ (Kozlovskaya et al., 1984; Grigoriev et al., 2004). For instance, according to Shenkman et al. (1997), the decrease in size of the slow and fast types of muscle fibers reached 15–18% by the 7th day of DI; similar changes were observed only after 60 days of HDBR. Very recent studies of cardiovascular, postural and neuromuscular changes under conditions of 21 days of HDBR and 3 days of DI revealed the similarity of their effects, suggesting that DI has an influence that is seven times stronger than HDBR (Tomilovskaya et al., 2018).

HDBR and DI reproduce the lack of axial loads, redistribution of body fluids in a cranial direction (to a greater extent in HDBR), hypokinesia and immobilization. The only substantial difference between these models is the level of support deprivation: in DI, support loads are totally eliminated, while HDBR provides the redistribution of support loads from the soles of the feet to the larger areas of the back and sides of the torso. The data from an experiment with combined exposure to HDBR and DI (alternating 12 h of HDBR and 12 h of DI) revealed significantly greater changes in periods of DI and their smoothing during HDBR sessions, confirming the leading role of support afferentation in the tonic postural system and motor acts regulation (Kozlovskaya et al., 1984).

Therefore, at the end of the 1970s, the DI model became the core component of studies focused on the effects of microgravity on the sensory-motor system. The motor system is considered to be the most gravity-dependent system of the human body. One of the main gravity-depended functions of the motor system is the maintenance of vertical posture and the position of body segments in the gravitational field. These functions are provided in mammals including humans by the tonic (postural) muscle system. The absence or sharp decline of gravity, eliminating the necessity of the aforementioned activities in weightlessness, greatly alters the functional and structural properties of tonic muscles. Orbital space flights have confirmed this suggestion and revealed the main factors which contribute to these effects of microgravity (Kozlovskaya, 2007). These factors are: a decrease of mechanical loads, a decline of axial and support loads, changes in the biomechanics of movements and changes in sensory system activities. The DI model reproduces accurately and to a full extent axial and support unloading, and significantly changes the sensory system’s activity. Exposure to DI eliminates the factor of support but at the same time does not directly affect the other sensory systems, which provides a definite advantage in comparison to other microgravity models and allows to study the role of support afferentation in the body systems’ activities.

In recent years the model of DI has gained popularity: a number of investigators from different countries (DLR in Germany, Angers University and Caen University in France) have taken part in the Russian DI research at IBMP. The results of these studies confirmed the supposition of the effects of support withdrawal on the activities of almost all body systems.

## DI Effects on the Neuromuscular System

Exposure to DI results in dramatic decline in muscle force-velocity properties, as well in muscle tone, and composition. Muscle effects, in particular a decrease in force-velocity properties, have been revealed even in very short term space flights (Kakurin et al., 1971; Bryanov et al., 1976; Kozlovskaya et al., 1984, 1988; Bachl et al., 1992); however, only under DI conditions their metrics and temporal dynamics could be studied in detail. Comparisons between the depth of changes after 7 days of DI and space flights of the same duration have revealed their close similarity (Grigorieva and Kozlovskaya, 1983; Kozlovskaya et al., 1984). DI experiments allowed not only to describe these phenomena but to get a better understanding about the nature of the decline in force-velocity properties of skeletal muscles (up to 30%), which cannot be explained by the muscle atrophy requiring much greater exposure times.

Subsequent studies of evoked and voluntary maximal contraction (MVC) strength of shin muscles confirmed this suggestion (Koryak, 1998, 1999, 2001). The results of the research have shown that MVC amplitude decreases similarly after 7 days of space flight, 7 days of DI and 4 months of HDBR. However, the amplitude of evoked responses declined insignificantly, indicating that the decrease of muscle force-velocity properties was caused by lower intensity of central efferent command (Koryak, 1998; Koryak, 2001).

One of the acute responses to transition to weightlessness is a flexor posture, which points to a decrease of the extensor muscle activity involved in the maintenance of vertical posture on Earth (Thornton, 1987; Reschke et al., 1998; Kozlovskaya, 2007). In fact, neurological observations performed immediately after short-term space flights in crew members of the first space expeditions revealed distinct extensor atonia (Bryanov et al., 1976). The results of studies of shin muscle transverse stiffness performed after 7-day space flights revealed a 15–20% decrease of muscle tone in m.gastrocnemius and m.soleus on the 2nd day after landing (Kozlovskaya et al., 1988).

This phenomenon was studied in detail in DI experiments on the ground, demonstrating the rapid decrease (up to 40–50%) of transverse stiffness in all three heads of the shin extensor — the lateral and medial m.gastrocnemius and most prominent – in m.soleus during the first 6 h of exposure (Gevlich et al., 1983; Kozlovskaya et al., 1988; Miller et al., 2004, 2005, 2010). The decrease of transverse stiffness of single muscle fibers was also revealed in m.soleus after 7-day DI (Ogneva et al., 2011). Studies of transverse stiffness in the shin flexor (m.tibialis anterior) did not reveal significant changes under DI conditions (Gevlich et al., 1983; Kozlovskaya et al., 1988; Miller et al., 2010).

Based on the above-mentioned data, the reflex nature of the muscle tone decline and its connection to the changes in motor units’ activity was suggested by Kirenskaya et al. (1986). This was confirmed in the studies of the recruitment order of motor units (MUs) in a task involving small effort plantar flexion (10–12% from maximal voluntary contraction) under conditions of 7 days of DI and 120 days of HDBR (Kirenskaya et al., 1986; Khristova et al., 1988; Kozlovskaya et al., 1988). The results of these studies allowed to conclude that support unloading causes the changes of recruitment order of shin extensor MUs through suppression of small (tonic) MUs recruitment and facilitation of recruitment of large (phasic) ones. Later, this suggestion was also confirmed in studies of DI of different duration (Shigueva et al., 2015b).

It is worth noting that revealed phenomena indicate that Henneman’s law of the MUs’ recruitment order is reversed under microgravity conditions. Classic Henneman’s law (or size principle) states that under load, motor units are recruited from smallest to largest. In practice, this means that slow-twitch, low-force, fatigue-resistant muscle fibers are activated before fast-twitch, high-force, less fatigue-resistant muscle fibers (Henneman et al., 1965). During DI, however, a motoneuron size is not the only factor defining the recruitment order.

In parallel to the neurophysiological research, a wide program of muscle morphological studies has been performed under DI conditions. Decreases in the size of both slow and fast types of muscle fibers reached 5–9% after 3 days and 15–18% after 7 days of DI (Shenkman et al., 1999; Shenkman, 2016; Shenkman et al., 2017). Meanwhile, the analogous changes after 60 days of HDBR reached 14.2 and 12.4% for slow and fast types of fibers, respectively (Shenkman et al., 2004a). Thus, the depth of size reduction of muscle fibers under conditions of short-term DI was commensurate to that under conditions of long term HDBR. This fact indicates the important role of support withdrawal in the genesis of hypogravitational muscle atrophy. It is important to note that the possibility of support-dependent control of atrophic processes has been discussed in a number of scientific papers (De-Doncker et al., 2000; Nemirovskaya and Shenkman, 2002; Ilyina-Kakueva and Kaplansky, 2005). However, this hypothesis was not validated in direct human experiments with stimulation of the support input. Oganov et al. (1980) first demonstrated the shift in the ratio of slow and fast myosin forms toward the fast ones under conditions of gravitational unloading, although these changes were not so evident in HDBR (Ilyina-Kakueva et al., 1979; Kuznetsov and Stepantsov, 1989, 1990; Berg, 1996).

More recent studies performed under strict conditions of maintenance of subjects’ supine position reported significant atrophy (up to 10%) of type I muscle fibers and increased portion of hybrid, typeI/II muscle fibers after 3 days of DI (Demangel et al., 2017). Molecular studies have also revealed changes in muscle signaling responses at the early stages of gravitational unloading (Vilchinskaya et al., 2015, 2016).

In summary, the neuromuscular effects of DI include:

1. Decrease in muscle force-velocity properties,
2. Decrease of tone in extensor muscles,
3. Suppression of small MUs recruitment and facilitation of recruitment of large ones,
4. Decreases in the size of both slow and fast types of muscle fibers.

## Does Support Withdrawal Triggers Hypogravitational Motor Syndrome Development?

The DI studies created a new branch in motor physiology research which can be termed as gravitational motor physiology. Studies of the functions and segments of the motor system both at the cellular and systemic levels have shown that gravity is deeply influential in the motor system. Primarily it affects muscles, or the effector system. The data from cell together with neurophysiological studies allowed scientists to suggest that gravity is a factor which affects the tonic postural system development. The deep skin sensitivity represented by clusters of Fatter-Paccini bodies is the trigger of this system which provides information on the presence or absence of gravity (Otelin et al., 1976).

**FIGURE 2:**
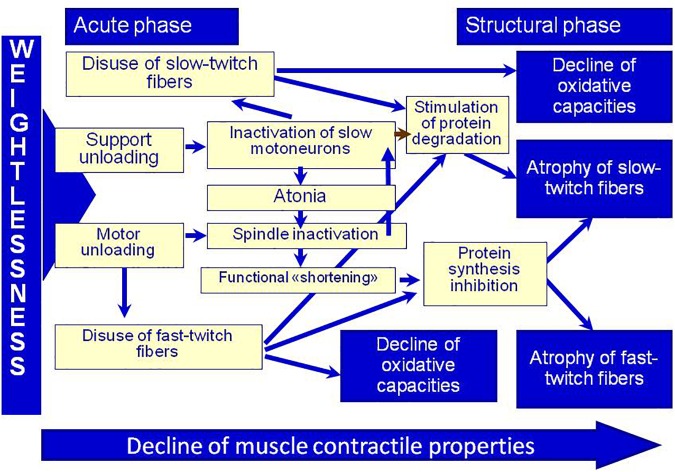
Physiological control of muscle plasticity in weightlessness (by Kozlovskaya I. B. and Shenkman B. S.). Adapted from Kozlovskaya, 2007.

Since there are no any other direct afferent changes in DI, support withdrawal is what promotes the sharp development of both cellular and systematic changes. This statement was illustrated by the scheme suggested by I. B. Kozlovskaya and B. S. Shenkman (Figure 2).

To confirm the trigger role of support afferentation in the control of the structural-functional properties of the tonic muscle system, the influence of mechanical stimulation of the soles support zones in locomotor regimens (75 and 120 steps per minute, pressure of 40 kPa in each sole) was tested. Stimulation was provided by Compensator of Support Unloading (CSU) developed jointly by IBMP, “Zvezda” and “VIT.” CSU provides alternating pressure of 0.5 kg/cm^2^ (40 kPa) on the support zones of the soles (heel and metatarsal areas).

The results of these studies confirmed the hypothesis that support afferentation plays the trigger role in the system of postural tonic control. The aforementioned changes in leg muscle functions were not observed in participants in whom the mechanical stimulation of the soles support zones during DI exposure was applied (Kozlovskaya et al., 2007a,b).

A decrease of transverse stiffness in m.soleus in this group reached significance only by the 6th day of DI; at the same time decrease of EMG amplitude at rest and maximal isokinetic force of m.triceps surae as well as signs of hyperreflexia and changes in MU recruitment order were not registered at all (Grigoriev et al., 2004; Khusnutdinova et al., 2004; Miller et al., 2004, 2005, 2010; Netreba et al., 2004, 2005, 2006; Kozlovskaya et al., 2007a,b; Shigueva et al., 2015a; Zakirova et al., 2015). Analogous effects of mechanical stimulation of the soles support zones were demonstrated in the structure of the muscular apparatus: no decrease of myofibrils’ sensitivity to free calcium ions, which is regularly observed under conditions of gravitational unloading; no changes in the cross sectional area of muscle fibers; and no decrease of the number of fibers containing slow isoforms of heavy myosin chains (Grigoriev et al., 2004; Moukhina et al., 2004; Shenkman et al., 2004a,b).

The natural consequence of the results described above was an offer to include the method of artificial support stimulation into the space flight countermeasure system for counteracting the early, acute motor system changes in weightlessness. Therefore, the device for mechanical stimulation of the soles support zones was modified in accordance with ISS requirements (the “CSU” device manufactured by IBMP and “Zvezda”), and at the end of 2017, it was delivered to the space station.

## DI Effects on the Sensory-Motor System

The DI studies have discovered that sensory-motor control systems are deeply affected when unloaded. First, the accuracy of movement control decreases (Repin, 1981; Kreidich et al., 1982; Grigorieva and Kozlovskaya, 1985; Kirenskaya et al., 1985; Sosnina et al., 2016). It has also been shown that the cortical organization of voluntary movements is changed (Kirenskaya et al., 2006; Tomilovskaya et al., 2008), and that the systems of posture and locomotion control suffer greatly (Melnik et al., 2006; Shpakov et al., 2008; Sayenko et al., 2016; Amirova et al., 2017). Similarly, studies of resting state brain activity revealed the changes similar to those obtained in a real space flight, indicating that support withdrawal is the main contributor to brain activity alterations and decline in the function of corticospinal tract function in weightlessness (Kuznetsova et al., 2015; Lazarev et al., 2018). Interestingly, support stimulation under DI conditions also eliminated these changes, although to a lesser extent than peripheral ones (Kozlovskaya et al., 2007a,b).

Vestibular function and visual-manual tracking is also significantly changed under DI conditions despite the absence of any direct influence on the vestibular system (Repin, 1981; Kreidich et al., 1982; Barmin et al., 1983; Kornilova et al., 2004, 2008, 2011a,b, 2013, 2016; Zobova et al., 2008).

For instance, in the studies of the static torsional otolith-cervical-ocular reflex (OCOR), dynamic vestibular-cervical-ocular reactions (VCOR), vestibular reactivity (VR), and spontaneous eye movements after 5- and 7-day DI a significant decrease in OCOR (gOCOR was 0.1, compared to the background gOCOR value of 0.25) was detected alongside a simultaneous significant increase in the VCOR/VR parameters in 28% of subjects on next day after DI. The findings have demonstrated that the support withdraw and the deficit of proprioception affected rather the accuracy of visual tracking than manual tracking. The obtained results confirm the development of sensory deprivation (and afferent deficit) under the DI exposure (Kornilova et al., 2016).

At the same time, the sensitivity to vestibular signals is sharply increased: the thresholds of oculographic response to galvanic stimulation decreased to 0.68–0.73 mA on the day 2 after 7-day DI in comparison to 0,92–1,05 mA before DI (Repin, 1981). Similar findings were observed in DI experiments performed in primates (Eron et al., 2000; Badakva et al., 2007). It is worth noting that no signs of motion sickness were reported during DI (Tomilovskaya, 2013b).

The sensitivity to proprioceptive signals was also changed: exposure to DI was associated with the development of H-reflex system hypersensitivity, which was demonstrated by a decrease of the soleus H-reflex thresholds and an increase of relative H-reflex amplitudes (Kozlovskaya et al., 2007a; Saenko, 2007; Zakirova et al., 2015).

As mentioned above, motion sickness was not observed during DI (Tomilovskaya, 2013b). Authors suggested that this may be due to rather motionless state of the participants. To test this, researchers began to investigate another type of water immersion – “suit immersion” (SI) — developed by IBMP specialists at the end of the 1980s (Genin et al., 1988a,b). SI, which used a thin-walled wetsuit “Forel” (“Trout” in Russian) equipped with an inflatable neck pillow to create postural, vestibular and operational loads directly during immersion, differed from DI by exclusion of strict hypokinesia and maintenance of vertical posture (Genin et al., 1988a,b; Larina et al., 1999; Larina and Lakota, 2000).

Although there is a difference in the body position between DI and SI, that is supine vs. vertical position, respectively, hemodynamics and water-salt exchange parameters have been shown to be similar (Genin et al., 1988a,b; Larina et al., 1999). However, during the first hours of SI, the development of vestibulo-autonomic syndrome, characterized by perception of body and head movements/rotations, was revealed (Genin et al., 1988b). In addition, the wide spectrum of illusions up to short-term loss of the awareness of body orientation were observed during SI. Following SI, a decrease of passive orthostatic probe tolerance, impaired postural control, an increase of EMG cost of standard motor tasks, and a significant decrease of physical and operator capacity were observed (Kreidich et al., 2007).

In summary, the effects of DI on the sensory-motor system include:

1. Decrease in accuracy of movement control,
2. Decline in descending regulation of voluntary movements,
3. Increased sensitivity to vestibular signals,
4. Decline in the accuracy of visual tracking,
5. Increased excitability of the soleus H-reflex.

## DI Effects on the Cardiorespiratory System

Studies of respiratory function have shown an increase of respiratory resistance, greater inspiratory and reduced expiratory reserve volumes, as well as changes in the thoracic-abdominal ratio in breathing motions and shifts in voluntary respiration regulation (Dyachenko et al., 2010; Mikhailovskaya et al., 2011; Popova et al., 2011). In addition, increased amplitude of breathing motions with reduced frequency has been noted, together with distinct breathing-related heart rate fluctuations (Semenov et al., 2011).

Furthermore, after 5 days of DI a significant increase in the markers of oxidative stress such as expired amines, chiefly butylamine, 2-cyanacetimide, some aldehydes, polyols, phenol, phenylacetylene, ketones, butyl acetate and a significant decrease in fatty acids has been observed (Mardanov et al., 2011). The mentioned changes indicate the alterations in metabolism and weakening of antioxidant system.

Studies of cardiovascular system changes in DI have revealed the subsequent involvement of electrical (increase of amplitude of QRS complex) and then – energy metabolic (decrease of heart rate and water-electrolyte balance alterations) processes changes in the myocardium; the most prominent changes were registered on the 5th day of DI (Ivanov et al., 2011). Autonomic regulation also appears to shift in the direction of the sympathetic component (Iwase et al., 2000; Eshmanova et al., 2008, 2009; Kabulova and Eshmanova, 2008).

Exposure to DI even of short duration (7 h) decreases orthostatic tolerance, and as the period of immersion is prolonged, the proportion of participants with orthostatic hypotension increases (Iwase et al., 2000; Vinogradova et al., 2002b; Eshmanova et al., 2009; Navasiolava et al., 2011a). All the studies revealed an increase in heart rate during vertical stance after DI of different duration (Navasiolava et al., 2011a,b). Recent studies performed after 3-days DI have shown decrease of systolic blood pressure, stroke volume and baroreflex sensitivity, pronounced tachycardia during low body negative pressure (LBNP) tilt test (De Abreu et al., 2017). The time of orthostatic tolerance in this study dropped from 27 to 9 min after DI; 9 of 12 subjects were unable to complete the test. Maximal LBNP value in the control studies reached -60 mmHg; 9 of 12 subjects have completed the test at the level of -30 mmHg. After DI exposure the LBNP level of -30 mmHg was reached by only 1 subject of 12 (De Abreu et al., 2017).

The relationship between changes in the water-electrolyte balance and cardiovascular responses revealed changes in electrophysiological propagation of myocardial excitation, and an increased variance of natural small oscillations of the electric potential of the heart (Larina et al., 2011). At the same time there was registered the increase of the brain natriuretic peptide BNP (as well as inactive NTproBNP secreted in an equimolar ratio), which is released by cardiomyocytes in response to stretching upon an increase in the ventricular pressure. The authors suggest that the increased level of NTproBNP during the recovery period following DI, reflects the degree of heart deconditioning during DI. The revealed intense tachycardia during orthostasis after DI, as well as the revealed changes in the electrophysiological characteristics of the myocardium and hypovolemia (one of the factors of development of the cardiovascular deconditioning under microgravity) during DI support the hypothesis of deconditioning induced increase in the heart load in the period of recovery. Within the first 24 h, the weight loss, rapid loss of fluid and sodium, and increase in the free cortisol content in urine were also observed. The changes in the water–electrolyte balance could cause metabolic–energy shifts that required activation of the respective regulatory mechanisms. A considerable (more than twofold) growth of the centralization index at the end of the experiment shows that inclusion of the central regulation mechanisms in the adaptation processes was an appropriate response aimed at compensation of the primary changes in the water–electrolyte balance and hemodynamics. The described changes appeared within the first 24 h of DI, and were manifested in the following days of the experiment. The changes in autonomic regulation and the electrophysiology of the myocardium during the experiment gradually increased, and reached maximum at the end of the 7th day of DI. Thus, it has been shown that the structures regulating the metabolic processes and, potentially, higher autonomic centers, from molecular–cellular to systemic levels, may be involved in the cardiorespiratory response occurring during the first 24 h of DI. The revealed alterations in the myocardial excitation warrants further research (Larina et al., 2011).

In summary, the effects of DI on the cardiorespiratory system include:

1. Increased respiratory resistance,
2. Greater inspiratory reserve volume
3. Reduced expiratory reserve volume,
4. Increase of the abdominal component during breathing,
5. Weakening of antioxidant system,
6. Increase of amplitude of QRS complex,
7. Decrease of heart rate,
8. Orthostatic hypotension,
9. Changes in the water-electrolyte balance and cardiovascular responses.

## Hemodynamic Changes in DI

Exposure to DI has also been associated with endothelial dysfunction, including an increase in circulating endothelial microparticles (Larina et al., 2008; Navasiolava et al., 2010, 2011b). Ultrasound analysis of peripheral hemodynamics have shown that the linear velocity of blood flow along main arteries and low extremity veins decelerates in the course of DI (Moreva, 2008; Moreva et al., 2018). At the same time, increased intensity of tissue perfusion and a higher number of capillaries in the upper extremities during 5-day DI were registered (Suvorov et al., 2017). Other authors, however, reported an increase of middle cerebral vein velocity and jugular vein, portal vein and thyroid volume, but these findings were obtained only during the first 2 h of DI (Arbeille et al., 2017). Another recent study failed to reveal changes in blood flow in either cerebral artery, but internal carotid and vertebral arteries conductance decreased significantly on the 2nd and 3rd day of DI (Ogoh et al., 2017).

It was also shown that DI significantly affects blood supply in working muscles. The peak bloodstream in the calf muscles decreases 7–20% after DI exposure, while post-contraction hyperemia noticeably increases (Vinogradova et al., 2002b). In studies of the blood flow of the tibia performed under conditions of physical loading, it was suggested that exposure to DI strengthened the concurrent relationships between local working hyperemia and central vasoconstrictive effects directed toward the maintenance of circulatory homeostasis (Stoida et al., 1998). Physical loading after 7-day DI was also followed by changes in energy and metabolism as indicated by decrease of creatine phosphokinase activity, and the changes of levels of cortisol, triglycerides, insulin and inorganic phosphate in blood plasma (Buravkova et al., 2003). However, no changes in metabolic reflex regulation of hemodynamic parameters under conditions of local static muscle work were registered (Bravy et al., 2008).

In summary, the effects of DI on hemodynamics include:

1. Endothelial dysfunction,
2. Decrease in the linear velocity of blood flow along main arteries and low extremity veins,
3. Decrease in blood supply of lower extremity muscles.

## DI Effects on Intracranial Pressure (ICP)

Since DI was proposed to reproduce distribution of body fluids similar to that during space flight, some research has been dedicated to the assessment of intracranial pressure. However, the data of ultrasound studies were characterized by high variability, and did not lead to definite conclusions (Arbeille et al., 2017). Use of the otoacoustic emission method through registration of electrical response of outer hair cells of the cochlea — DPMC (Phase Shift of Microphonic Cochlear Potential) — showed that body position and DI do affect the DPMC (and thus ICP). Changes in ICP were similar during a supine-to-DI position change and supine-to-antiorthostatic position change. Following DI, a tendency to decreased phase shift of the otoacoustic response during transition of the body position from a vertical to antiorthostatic one, was revealed, which could be associated with the ICP increase (Avan et al., 2013; Rukavishnikov et al., 2013).

A study of optic nerve sheath diameter (ONSD) and cerebral autoregulation revealed a strong negative correlation between these two parameters. The authors suggested that a persistent elevation of ICP can be attributed to poor cerebral autoregulation recovery during DI (Kermorgant et al., 2017).

## DI Effects on Renal and Digestive Systems

Studies of the excretory function of the kidneys together with the classical increase of dieresis revealed an increase of protein and glucose excretion, although not exceeding physiological standards (Vorontsov et al., 2011, 2014). No shifts of glomerular filtration or tubular reabsorption were registered (Nesterovskaya et al., 2008). At the same time, the impedance measurement revealed a decrease of general body fluid and the volume of extracellular fluid as well as the volume of circulating plasma (Larina et al., 2008; Noskov et al., 2011).

Other studies report that exposure to DI causes rapid fluid and sodium loss, which increases the Na/K ratio in urine. The accompanying body weight loss is due to a decrease in the level of body hydration. An increase in the level of free cortisol in urine only on the 1st day of DI may be due to adaptation to the new conditions. The most pronounced changes were observed on the 1st day of DI. On the following days, a new level of water–electrolyte balance is established. The absence of changes in the renin or aldosterone level on days 3 and 7 was evidence that the major redistribution of fluids had been completed by that time and regulation of the water–electrolyte balance had stabilized. The decrease in urine osmolality during DI characterizes an enhanced excretion of water but not electrolytes and shows that the regulatory mechanisms perceive water consumption during DI as excessive. In addition, this suggests a decrease in the concentration capacity of the kidneys (Larina et al., 2011).

Studies of the digestive system have revealed deceleration in the hepatic venous flow and signs of plethora in the abdominal venous system (Solovieva et al., 2016). Elevated levels of several substances were detected in the blood: pepsinogen, pancreatic amylase, bilirubin total (due to its unconjugated fraction), insulin, and C-peptide. The 13C-methacetin breath test has shown a slowdown in the rate of 13C-methacetin inactivation and a reduction in the hepatic metabolic capacity (Solovieva et al., 2016). The study of the liquid food evacuation during immersion has shown that its rate did not change substantially, therefore, reductions in the rates of methacetin inactivation and metabolism during immersion could be associated, mainly, with a slower assimilation of the preparation and a decrease in the venous blood flow of the liver. A reduction in the metabolic capacity of the liver in the conditions of immersion reflects a decrease in the inactivation of toxic metabolites, including unconjugated bilirubin, which may explain the increase of its content in blood.

Further studies have demonstrated the stability of liquid evacuation from the stomach and acceleration of the chymus transit along the small intestine hinder evacuation of the large intestine content, which is the primary cause for inhibition of gastrointestinal evacuatory activity in DI (Afonin and Goncharova, 2009; Afonin et al., 2013). The elevated gastrointestinal electrical activity may be related to the increased gastric secretion and elevated intestine tone in fasting test subjects, and displayed a close similarity to the changes induced by caffeine stimulation, long-term bed rest or space flight (Afonin and Sedova, 2012). At the same time, clear signs of dysbiotic changes such as significant reduce of fecal lactoflora and developed shifts in the microbial landscape (increase of the number of staphylococcus, yeast microflora and Gram-negative Bacillus) have been revealed (Ilyin et al., 2008; Solovieva et al., 2011).

In summary, the effects of DI on the renal and digestive systems include:

1. Decrease of general body fluid and the volume of extracellular fluid as well as the volume of circulating plasma,
2. Changes associated with rapid fluid and sodium loss,
3. Changes in the hepatic and the abdominal venous systems,
4. Reduction in the metabolic capacity of the liver,
5. Inhibition of gastrointestinal evacuatory activity in DI,
6. Dysbiotic changes.

## DI Effects on Biochemical Parameters of Blood and Urine

Biochemical tests revealed changes in blood and urine protein content (Trifonova et al., 2010; Pastushkova et al., 2011; Vorontsov et al., 2014). Specifically, there is a decrease of muscle and cardiac constellation ferments activity, increase of lipid metabolism derivatives, increase of erythrocytes concentration, decrease in glucose tolerance and hypercholesterolemia, and increase of insulin and serum NT-proBNP levels, which is a proxy measure for brain natriuretic peptide (Ivanova et al., 2008; Markin et al., 2008; Navasiolava et al., 2011c; Coupe et al., 2013; Pastushkova et al., 2014; De Abreu et al., 2017). The new series of DI studies introduced the analysis of blood plasma proteome (Pastushkova et al., 2012). On DI days 2 and 3, growth of peak areas was observed in fragments of complement system proteins C3 and C4, high-molecular kininogen and fibrinogen. Significant increases of the peak area of apolipoprotein CI (reduced form with segregated threonine and proline) and C4 enzymes of the complement system, and fibrinogen on the 1st day after the experiment can be related to changes in motor activities of the subjects.

Analysis of the hemostasis system did not reveal any significant changes, although a tendency in decrease of antithrombin III activity (ATIII), protein C and plasminogen after exposure to 7-day DI was observed (Kuzichkin et al., 2010). Studies of antioxidant protection and lipid peroxidation rates did not reveal changes in their values during adaptation to DI. However, the return to normal activity after DI was associated with the development of a significant stress reaction, as evidenced by the strengthening of the lipoperoxidation, and a decrease in the functional activity of the antioxidant defense system (Zhuravleva et al., 2012).

In summary, the effects of DI on the biochemical composition of blood include: changes in the concentration of protein and lipid derivatives.

## DI Effects on Skeletal System

It is not surprising that there are not many studies dedicated to bone marker changes in DI, given that the skeletal system is quite inert and changes occurring throughout a relatively short exposure to DI, such as 7 days, are not expected. There were no effects of 7-day DI on bone formation or increase of bone resorption in the study of Kopp (2008). A recent study performed during 3-day DI, however, revealed significant decrement of bone formation markers, namely total procollagen type I N- and C-terminal propetides and osteoprotegerin (Linossier et al., 2017).

## DI Effects on Immune System

Studies of the DI effects on innate and acquired immunity revealed high variability of changes during DI (Berendeeva et al., 2011; Ponomarev et al., 2013). Observed negative shifts in congenital immunity at the end of 5-day DI and during recovery, suggest that there is an increased risk of infectious diseases, for instance caused by changes in normal microflora which normally does not cause pathological effects (Ponomarev et al., 2013). There were changes in the content of immunoglobulin (sIgA, IgA, and IgM) in the gingival fluid, which can reflect inflammation process in the periodontal tissue. Presence in the oral cavity of periodontopathogenic type of bacteria, namely, *Prevotella intermedia*, *Tannerella forsythia*, *Treponema denticola*, *Aggregatibacter actinomycetemcomitans*, *Porphyromonas gingivalis*, was also noted (Nosovsky et al., 2017).

A study of the neuroendocrine regulation during DI has demonstrated a decrease of triiodothyronine and cortisol levels, as well as an increase of prolactin and thyroxine concentration (Nichiporuk et al., 2011; De Abreu et al., 2017). The rate of changes in immunity parameters among different subjects demonstrated the highest frequency in non-anxious and extravert individuals on day-5 in DI (Nichiporuk et al., 2011).

## Effects of Di on Thermoregulation

Exposure to short and long-term space flights is followed by gradual increase of core body temperature by 1°C over 2.5 months of flight, which was suggested to be associated with augmented concentrations of interleukin-1 receptor antagonist, a key anti-inflammatory protein (Stahn et al., 2017). Unfortunately, there no published studies on thermoregulation during DI, except for one report on the lack of significant changes in the body temperature on the day 3 of DI (De Abreu et al., 2017).

## DI Results in the Back Pain Phenomenon

**FIGURE 3:**
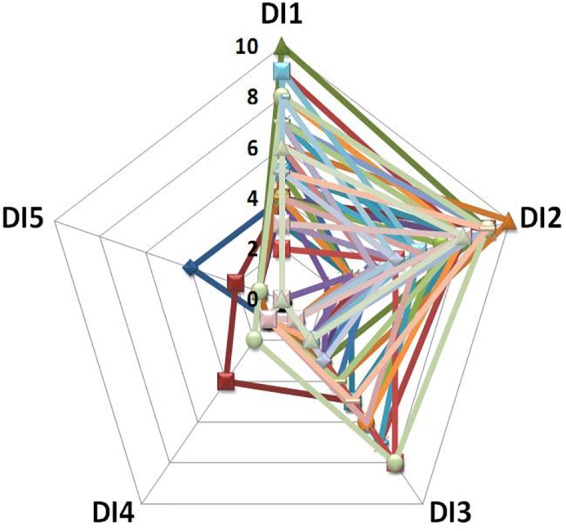
Intensity and duration of back pain during DI: data from 39 participants (Rukavishnikov et al., 2014b). *X*-axis: day of DI; *Y*-axis: pain intensity on a 10-point subjective scale.

A recent series of DI experiments explored the potential mechanisms of the back pain which is regularly observed at the beginning of space missions and under ground-based conditions. Almost all DI study participants report back pain during the first 2–3 days of DI (Rukavishnikov et al., 2014a,b, 2015, 2017a,b; Kozlovskaya et al., 2015; Treffel et al., 2017). Retrospective analysis of the data from nine of the most recent DI experiments conducted at IBMP (2006–2018) demonstrated that 70 out of 87 participants reported back pain with an intensity ranging from 4–5 to 9–10 points on a ten-mark subjective scale (see Figure 3) (Rukavishnikov et al., 2014b).

The findings also revealed a decrease of back extensor transverse stiffness (Rukavishnikov et al., 2017a,b, 2018). Data obtained during magnetic resonance imaging (MRI) also showed that vertebral column length significantly increased in the neck, thoracic and lumbar parts of the spine (Kozlovskaya et al., 2015; Rukavishnikov et al., 2015; Treffel et al., 2016a,b). At the same time, the height of intervertebral disks increased, the disks swelling was observed, the neck and thoracic kyphosis were flattened, as were the thoracic and lumbar lordosis. MRI analysis revealed a decreased cross sectional area of mm. quadratus lumborum, multifidus, and erector spinae at the level of L4–L5 (Rukavishnikov et al., 2017b). It has been proposed that each of these factors can contribute to the hypogravitational back pain, including intervertebral disk swelling, spine lengthening, influence on the yellow ligament of the spine and back muscle atonia (Treffel et al., 2016b; Rukavishnikov et al., 2017b).

## Contribution of DI Model to Space Flight Countermeasure System Development

As indicated at the beginning of this review, DI has been developed to investigate in depth the mechanisms of the effects of microgravity on the human body. Logically, DI has become instrumental in the development of a countermeasure system to prevent negative physiological effects of space flights. The history and extent of the studies employing the DI model and leading to the tests, comparison, and optimization of various countermeasure approaches, worth to be discussed in a separate review. In this section, we focus on the major outcomes which originated the design of the current countermeasure programs for use on board the International Space Station.

In the late 1970s, studies of short arm centrifuge were performed (Shulzhenko and Vill-Villiams, 1976; Grigoriev and Shulzhenko, 1979; Vil-Villiams and Shulzhenko, 1980; Clement and Pavy-Le Traon, 2004). The experiments performed under conditions of 13- and 56-day DI have shown that application of short arm centrifuge (SAC) 0,5–1 Gz rotations in the course of DI was followed by maintenance of back-up capabilities of cardiovascular system and external respiration and increase of resistance to 5 min +3 Gz rotations in “head-pelvis” direction. Combination of SAC sessions with water-salt supplements and 2340 kg physical loads (hand and leg exercises with expanders) increased countermeasure effects in this case (Shulzhenko and Vill-Villiams, 1976; Kokova, 1983).

Later, the number of DI studies aimed to examine the effects of different countermeasure means were performed. For instance, the studies of the efficacy of antigravity suit of bladderless type in maintenance of orthostatic tolerance have been carried out under conditions of 7-day DI (Shulzhenko et al., 1983b; Vil-Villiams et al., 1996). With the suit “Centaur” on, the 20 min tilt test at 70 degrees induced less changes in blood pressure as compared with that without the suit. The maximum heart rate significantly decreased, minimum stroke volume and pulse pressure increased. As such, using DI model the authors revealed that the anti-G suit helps to increase orthostatic tolerance and can be used following space flights.

Early DI studies demonstrated that the application of 25 mm Hg negative pressure to the lower body (LBNP) can counteract the decrease in the orthostatic tolerance (Genin and Pestov, 1974). The water-salt additives combined with LBNP and physical training, increased the capacity of vascular bed and help to retain body fluids, sodium, and chloride during DI (Grigoriev and Shulzhenko, 1979).

The positive effect of hip occlusion cuffs in preventing orthostatic intolerance after 18-h immersion strongly depended on the duration of break between occlusion sessions: the best effect has been demonstrated in the case of 1-min break (the pressure consisted 25 mm Hg, the duration of training session – 7 h) (Genin and Pestov, 1974). The mechanisms of the countermeasure effects of occlusion cuffs revealed in DI experiments, are based on the pooling of blood in vessels of the lower extremities due to the occlusion of superficial veins and hence – decrease of blood volume that is shifted in cranial direction (At’kov and Bednenko, 1987).

During 28-day DI study, the efficacy of isolated and combined effects of SAC and veloergometry have been examined. The study has shown that veloergometry training in the regimen of 600 kg/min (10 min training - 10 min break) for 60 min twice a day was followed by increase of cardiac input and decrease of total peripheral resistance. Combination of veloergometry training with SAC (0,8–1,9 Hz twice a day for 60 min per session) enforced the countermeasure effect which was assessed by cardio-vascular system parameters (Vil-Villiams and Shulzhenko, 1980; Vil-Villiams, 1994).

Effects of low and high-frequency electrostimulation of leg muscles have been studied in the series of DI experiments (Koryak et al., 2008; Kozlovskaya, 2008; Eshmanova et al., 2009; Koryak, 2014, 2018; Solovieva et al., 2016). These studies demonstrated that daily use of low frequency stimulation can counteract the decrease of force-velocity properties in m. triceps surae, especially when the intensity of stimulation is rather high (more than 13 V). Comparative evaluation of the efficacy of low-frequency stimulation of the lower limb muscles versus high frequency stimulation during 7-days DI, showed superiority of the low-frequency stimulation in prevention of motor dysfunctions (Koryak et al., 2008; Kozlovskaya, 2008). During these experiments, positive effects of neuromuscular electrical stimulation were also associated with unloading of the right heart and revealed by HD ECG parameters and growth of the “myocardium” index (Eshmanova et al., 2009). The application of high frequency electromyostimulation prevented elevation in pepsinogen, pancreatic amylase, and bilirubin, predominantly within its unconjugated fraction, as well as an increase in insulin secretion; however, it did not affect the ultrasound patterns of the hemodynamic rearrangement in both the liver and the abdomen (Solovieva et al., 2016).

A wide program of DI studies was dedicated to the effects of mechanical stimulation of the plantar mechanoreceptors in locomotor regimens. These studies confirmed the hypothesis on the trigger role of support (weight bearing) afferentation in the hypogravitational motor syndrome development (Grigoriev et al., 2004; Kozlovskaya et al., 2007b; Layne and Forth, 2008). Application of the plantar mechanostimulation during six 20-min sessions per day (with the stimulation frequency of 75 and 120 steps/min and pressure 40 kPa) eliminated the negative effects of gravitational unloading on sensory-motor and neuromuscular systems, including the decline of extensor muscle stiffness and decrease of the maximal voluntary force; a significant decrease of the absolute force of the isometric contraction of single skinned muscle fibers; a prominent decline of the tonic muscle fibers transversal size; and the transformation of the myosin phenotype from slow to fast one (Popov et al., 2003; Grigoriev et al., 2004; Khusnutdinova et al., 2004; Litvinova et al., 2004; Miller et al., 2004; Moukhina et al., 2004; Shenkman et al., 2004a,b; Netreba et al., 2005; Kozlovskaya et al., 2007a,b). The results of studies of biomechanical characteristics of locomotions brought to the conclusion that the rates of mechanical foot stimulation applied in the experiment did not change energy expenditure in the muscles; however, they moderated the amplitude of angular knee joint movements following 7 days of DI (Melnik et al., 2006). Application of the support stimulation was also effective in preventing venous compliance increase and orthostatic intolerance (Vinogradova et al., 2002a).

Countermeasure effects of the soles support zones stimulation has been also demonstrated in the visual-vestibular function. The group of subjects without support stimulation exhibited marked omnidirectional deviations in the eye tracking parameters, whereas in the group of subjects with support stimulation, these parameters were similar to the baseline values. Support stimulation also stabilized the pursuit function of the eye making it less variant. However, there was no uniformity in the subjects’ reaction to stimulation, which infers that the methods of improving the eye pursuit function should be personally “molded” (Kornilova et al., 2004).

Rather few data can be found in the literature concerning the studies of countermeasure effects of different pharmacological means with use of DI model. In the studies of pain sensitivity under conditions of DI, no reduction in the morning pain sensitivity (which is typical for normal conditions) has been revealed. Such analgesic as ketorolac had no effect on pain sensitivity, when determining the pain threshold by method of thermo algometry. The authors noticed that DI substantially altered the pharmacokinetics of ketorolac, increasing the rate of absorption of the drug and reduction of its relative bioavailability and retention time in the blood. These results indicate that pain therapy schemes may be administered differently during space flight as compared with 1G (Baranov et al., 2015).

In 7-day DI experiments the pharyngeal microflora in 22 healthy volunteers has been studied. For prophylactic pharyngeal dysbiosis, two probiotic drugs were used: oral - “lactobacterin dry” and local -“lactobacterin immobilized on collagen.” Administration of oral probiotic was accompanied with growth of pharyngeal opportunistic microflora preventing translocation of intestinal microflora. Local probiotic, on the contrary, decreased opportunistic microflora in the pharynx, but was associated with gastrointestinal dysbiosis. It was concluded that the combination of topic and oral probiotics provides the maximally effective prophylaxis of pharyngeal dysbiosis during DI (Kryukov et al., 2007; Ilyin et al., 2008). In another 7-DI study, the effects of sydnocarb [3-(beta-phenylisopropyl)-*N*-phenyl carbamoyl sydnonimine] were investigated. The subjects exercised on a bicycle ergometer before and after water immersion. During exercises, ECG, heart rate, minute respiration volume, oxygen consumption, carbon dioxide production, cardiac output, oxygen pulse were recorded. The subjects who received placebo showed a significant decrease of oxygen consumption at a maximum workload. Those who were given sydnocarb maintained normal oxygen consumption during bicycle ergometry. The drug increased the workload per kg body weight, maintained physical work capacity, and improved the cardiovascular function after DI exposure (Anashkin and Belyaev, 1982). The positive effect of the pharmaceutical – alleviation of myocardium strain – was a result of amlodipin (slow calcium channel inhibitor) assistance to the coronary blood flow; however, it had a negative side-effect on orthostatic stability (Eshmanova et al., 2009).

The DI model was actively used during the development of the “Penguin” axial loading suit (Barer et al., 1998). The studies have revealed increased EMG amplitude in the lower extremities muscles in subjects who wore Penguin suit while exercising on a bicycle ergometer. The same studies revealed increased heart rate and metabolic rate values in the subjects during performing the step loading veloergometric test with Penguin use. Other DI studies have shown that the “Penguin” suit was effective in preventing hypogravitational hyperreflexia, peripheral microcirculation changes, height increase, back pain development and back muscle atonia, and a decrease in force-velocity properties (Barer et al., 1998; Shigueva et al., 2015b; Rukavishnikov et al., 2015; Suvorov et al., 2017).

## Contribution of DI Model to the Terrestrial Medicine

The findings on the physiological effects of DI on various systems of the human body, found their clinical application starting from the 1980s. First, the effects of DI in muscle relaxation, decreasing skeletal muscles tone and eliminating muscle spasticity, were noted (Kozlovskaya I. B. and Semenova K. A., unpublished observations).

The DI has been applied in edema therapy, especially intractable ones induced by cardiovascular etiologies as well as by burns, liver cirrhosis, and renal disease. In individuals with the mentioned syndromes, the effects of 1.5–3 times diuresis increase after 4–6 h post DI was maintained during 2 days (Yunusov et al., 1985; Ivanov and Bogomazov, 1988).

During the 1990s, several studies utilized DI in therapy of hypertensive crisis (Kirichenko et al., 1988; Evdokimova et al., 1989; Ivanov and Makarova, 1990). The results revealed a significant and persistent hypotensive effect after 90 min of exposure to DI. The effects were pronounced through two different mechanisms: (a) via a decrease of elevated total peripheral vascular resistance (TPVR) and no changes in cardiac output, or (b) via a decrease of elevated cardiac output. These hemodynamic effects were attributed to sanogenetic mechanisms which optimize blood circulation during hypertension (Evdokimova et al., 1989).

Recent studies (Meigal et al., 2017) have revealed positive effect of short-term, up to 6 h, DI sessions in patients with either Parkinson’s disease or parkinsonism: the score of subscale I of Wein’s inventory for autonomic disorders has decreased by 45%, and of the subscale II – by 23%. In addition, the time of the choice reaction has significantly improved. These effects were also observed 2 weeks post the course of DI.

The DI is widely used in pediatrics as a component of rehabilitation programs for premature children, to normalize hemodynamics and prevent brain edema. Exposure to DI is also used for the rehabilitation of children with central nervous system disorders, such as perinatal encephalopathy and other perinatal pathology (Kazanskaya, 2008; Khan et al., 2015). The method is also widely used in children undergoing cerebral palsy therapy, since it is effective in lowering hypertonus and hyperactivity, and in reflex facilitation (Yatsuk, 2002; Titarenko et al., 2015).

On the contrary to negative effects of 3–7 days DI on immune system (see above), the positive effect of 40–50 min DI on immunological parameters were revealed. Namely, promoted normalization of the functional activity of T- helpers and the lower adhesive properties of lymphocytes, as well as reduction in the incidence and severity of neonatal infectious and inflammatory diseases have been demonstrated (Chasha et al., 2008).

The diagnostic value of DI in the early detection of neurological pathologies under normal conditions was suggested in joint Russian–Austrian experiments. After 24, 48, and 72 h of DI, some healthy participants showed neurological symptoms (such as cerebellar disintegration, deterioration in the function of peripheral nerves, posterior tract, as well as pyramid and extrapyramidal systems) (Gerstenbrand and Kozlovskaya, 1990; Berger et al., 2001). This phenomenon may be explained by the fact that some people have latent neurological deficiency (congenital or acquired as a result of injuries, infections, etc.), but the central nervous system’s high plasticity typically provides compensation for these conditions. The sensory conflict created by DI (changing the support and proprioceptive afferent inflow) removes compensatory mechanisms that are ineffective in the new conditions, and hidden neurological disorders are revealed.

Due to its relaxation effects, DI was also used in sports medicine for recovery of athletes after high intensity training (Radzievskiy and Radziyevskaya, 2007).

There are also medical contraindications to the use of DI. According to various experts, they include myocardial infarction, severe cardiac arrhythmia, chronic respiratory diseases with pulmonary heart decompensation, thrombophlebitis, severe shortness of breath at rest (Ivanov and Makarova, 1990), acute inflammatory processes and generalized lesions of the skin (Yatsuk, 2002). However, these considerations are mostly theoretical and no quantitative results have been reported.

## Conclusion

Dry immersion reproduces to some extent many of the changes associated with spaceflight. DI can serve as a valid ground-based model for simulation of physiological effects of weightlessness for some systems. The magnitude and rate of physiological changes during DI makes this method advantageous as compared with other ground-based microgravity models. DI is instrumental in the design of countermeasure programs for use in space, especially, for passive countermeasures which do not require intensive motor activity of the subjects. DI may also have a range of clinical applications that warrant further study.

## Author Contributions

ET wrote the draft of the manuscript and made its revisions. TS prepared the part on DI effects in sensory-motor system and designed the figures. DS contributed in the global revision and reorganizing of the manuscript. IR prepared the part on back pain phenomenon and implementation of the DI model in Earth medicine. IK made the revision of the manuscript and prepared the part of use of DI for countermeasures development.

## Conflict of Interest Statement

The authors declare that the research was conducted in the absence of any commercial or financial relationships that could be construed as a potential conflict of interest.
